# Silver Conductive Adhesives with Long Pot Life and Stable Electrical–Thermal Performance

**DOI:** 10.3390/polym18080899

**Published:** 2026-04-08

**Authors:** Wilson Hou-Sheng Huang, Jyh-Ferng Yang, Yi-Cang Lai, Jem-Kun Chen

**Affiliations:** 1 Center for Continuing Education, National Tsing Hua University, Hsinchu 30013, Taiwan; 2 Department of Chemical Engineering, I-Shou University, Kaohsiung 84001, Taiwan; 3 Department of Materials Science and Engineering, National Taiwan University of Science and Technology, Taipei 106335, Taiwan

**Keywords:** silver conductive adhesive, low temperature curing, pot life, volume resistivity, flexible substrates

## Abstract

This study systematically investigates the formulation–property relationships of epoxy-based silver conductive adhesives by varying silver filler architecture, total filler loading, and organic carrier design. Rotational viscometry, four-point probe measurements, thermal conductivity analysis, and scanning electron microscopy (SEM) were employed to elucidate the correlations among rheological behavior, conductive network formation, and electrical–thermal transport properties. All formulations incorporate dicyandiamide (DICY) as a latent curing agent, in combination with a thermally activated accelerator and silane coupling agents, to stabilize filler–matrix interfaces and suppress moisture-assisted side reactions. This latent curing chemistry enables effective low temperature curing at approximately 155 °C, providing compatibility with temperature-sensitive flexible polymer substrates. After sealed storage at 25 °C and 60% relative humidity for two weeks, all formulations exhibited viscosity variations within ≤16%, demonstrating extended pot life and good storage stability under ambient conditions. Meanwhile, the normalized volume resistivity and thermal conductivity remained close to their initial values, with maximum relative deviations of approximately 12% and 7%, respectively, from the initial (Day 0) values across all formulations, indicating stable electrical and thermal transport properties during storage. Differences in conductive network formation and filler packing characteristics were reflected in the observed electrical and thermal transport behaviors. Balanced electrical–thermal performance was achieved without the need for high-temperature sintering or post-annealing, underscoring the effectiveness of the low temperature curing strategy. Overall, this work defines a practical formulation design window that simultaneously achieves low temperature curability, long pot life, stable rheology, and robust electrical–thermal performance. The results provide useful material-level guidelines for the development of epoxy-based silver conductive adhesives intended for conductive interconnects on flexible polymer substrates and related flexible electronic applications.

## 1. Introduction

The performance of silver-based conductive adhesives (ICAs) emerges from the interplay between metallic filler characteristics and the surrounding polymer matrix, as mediated by curing chemistry and interfacial design. While epoxy formulation and curing behavior establish the processing window, the formation and durability of electrically conductive pathways are predominantly governed by the geometric, chemical, and interfacial attributes of silver fillers. Previous studies have highlighted the critical role of filler design in determining electrical and thermal performance. For instance, Song et al. [1] demonstrated that in situ-formed silver nanoparticles can significantly enhance the electrical properties of epoxy systems by improving conductive network formation. Tseng et al. [2] adopted a molecular design strategy to enhance thermal conductivity in conductive adhesives, emphasizing the importance of interfacial interactions. In addition, Jin et al. [3] showed that post-treatment of silver nanowire/polymer composites can improve electrical performance through better interparticle contact. Furthermore, Kumar et al. [4] reported that hybrid filler systems, such as expanded graphite combined with silver flakes, can effectively improve thermal transport properties. These studies collectively indicate that filler morphology, surface condition, and interfacial engineering play essential roles in governing interparticle contacts and network continuity, thereby determining both the initial electrical conductivity and its long-term stability. Importantly, charge transport in ICAs does not scale solely with the intrinsic conductivity of silver, but is frequently limited by constriction and contact resistance at particle–particle junctions, which evolve dynamically during curing and post-curing densification. For example, Cui et al. [5] demonstrated that interparticle contact and filler–filler interactions play a dominant role in determining electrical conductivity, while Zhang et al. [6] showed that the construction of multiscale conductive bridge structures can improve conductive network efficiency and enhance charge transport. Other studies have also reported related behaviors under different material systems and processing conditions [7,8].

Rheology plays an equally crucial role in determining the processability and microstructural evolution of silver adhesives. The viscosity and thixotropic recovery of the organic vehicle determine not only the suspension stability of fillers but also the fidelity of deposited patterns during screen printing, dispensing, or stencil printing. For example, Thibert et al. [9] and Lee et al. [10] demonstrated that paste rheology strongly influences printing resolution and pattern fidelity, while Nam et al. [11] and Hu et al. [12] emphasized the importance of paste formulation and rheological properties in maintaining suspension stability and processability. Organic carriers based on cellulose derivatives, phenoxy resins, thermoplastic modifiers, or mixed-solvent systems have been designed to tailor flow behavior, leveling, and structural recovery. Bari et al. reported that slight variations in solvent polarity or evaporation rate could strongly influence filler rearrangement during the gel-to-solid transition, ultimately affecting network uniformity and conductivity [13].

Other studies have shown that surfactant architecture and dispersant molecular design play pivotal roles in preventing agglomeration and ensuring homogeneous filler distribution. For example, Kosmala et al. [14] demonstrated the importance of stabilizing highly concentrated silver dispersions, while ligand engineering strategies have been shown to further improve dispersion stability and processability [15,16]. In dense metallic adhesives, these subtle rheological deviations during early-stage curing can yield measurable differences in conductive pathway formation.

Interfacial chemistry further governs the performance and reliability of ICAs. Surface modification strategies, including silane coupling and thiol functionalization, have been widely employed to tailor interfacial interactions and improve adhesion at the silver–epoxy interface [17,18]. In addition, the incorporation of nanostructured components and interfacial design strategies has been shown to enhance filler dispersion and facilitate conductive network formation, thereby improving the overall electrical and thermal performance of the composites [19,20]. For example, when silver nanoparticles are modified by silane coupling agents prior to incorporation into epoxy, improved filler surface chemistry leads to better dispersion and enhanced conductive-adhesive performance [21]. In contrast, environmental exposure, particularly humidity, can induce chemical modifications at silver surfaces, resulting in oxide formation, interfacial degradation, and progressive increases in electrical resistance during accelerated aging [22,23]. These findings collectively highlight the importance of interfacial engineering for maintaining long-term electrical stability.

In practical applications, another critical challenge associated with epoxy-based silver conductive adhesives is the control of pot life, which is defined here as the viscosity stability of the formulation during ambient storage. Premature viscosity increase during storage is commonly associated with residual catalytic species or reactive accelerators remaining in the formulation. These species can trigger epoxy homopolymerization even below the intended curing temperature, leading to the gradual formation of early-stage polymerization products or partially crosslinked structures within the organic vehicle [24]. As a consequence, the progressive increase in molecular weight during storage results in a pronounced rise in viscosity, thereby significantly reducing the processing window and processability of the formulation [25]. In addition, formulation strategies such as one-component epoxy systems typically involve latent curing agents or catalytic species that must be carefully controlled to balance storage stability and curing reactivity [26]. Latent curing systems have been widely developed to delay curing activation and improve storage stability in epoxy formulations. Among them, DICY is a widely used latent curing agent for epoxy-based adhesives and coatings [27]. Epoxy systems cured with DICY are widely used in practical applications such as printed circuit board fabrication [28]. In particular, microencapsulated imidazole curing systems have been shown to suppress premature reactions during storage and improve storage stability, while still enabling curing upon thermal activation [29]. Furthermore, interactions between curing agents, fillers, and interfacial modifiers may influence curing behavior and network formation. Previous studies have shown that the presence of filler surfaces or functional interfaces can influence curing behavior and network formation, thereby affecting the final electrical and mechanical performance of conductive adhesives [30,31]. Recent studies have further highlighted the importance of interfacial chemistry in conductive systems. Jeon et al. demonstrated that introducing an amine-functional self-assembled monolayer significantly alters silver nucleation behavior and improves the uniformity and adhesion of printed Ag electrodes, underscoring the critical role of interfacial design in determining electronic performance [32].

However, the coupling effects among latent curing behavior, filler packing characteristics, and vehicle rheology are still underexplored, especially at high silver contents where particle jamming and early-stage viscosity evolution become dominant factors. Meanwhile, the rapid expansion of flexible electronics, high power semiconductor modules, photovoltaic metallization, mini-LED packaging, and energy device manufacturing has generated growing demand for conductive adhesives that simultaneously deliver high conductivity, strong adhesion, controlled thixotropy, thermal robustness, and long pot life [33,34].

Motivated by the above considerations, this study systematically investigates the effects of silver filler characteristics, total filler loading, and latent curing chemistry on the performance of epoxy-based silver conductive adhesives. DICY is employed as a latent curing agent to achieve extended pot life and stable viscosity during storage, while simultaneously enabling low temperature curing compatible with the processing constraints of wearable electronics and flexible substrates. In addition, silane coupling agents are incorporated to regulate interfacial interactions between silver fillers and the epoxy matrix, thereby enhancing interfacial bonding and suppressing moisture-induced interfacial degradation. By comparing different commercial silver fillers under identical organic vehicle compositions and curing conditions, this work aims to elucidate the roles of filler characteristics in governing electron transport, thermal conduction, and rheological behavior. The resulting insights provide systematic formulation guidelines for conductive adhesives that combine low electrical resistivity, high thermal conductivity, favorable thixotropic behavior, low temperature process compatibility, and long-term storage stability, which are essential for wearable electronic devices, flexible interconnect architectures, and high power electronic packaging applications.

## 2. Materials and Methods

### 2.1. Materials

Silver flakes (SA-4391 and SA-4084) were purchased from Metalor Technologies (Marin-Epagnier, Switzerland). The specific surface areas of SA-4391 and SA-4084 were 1.00 m^2^/g and 0.76 m^2^/g, respectively. DICY was obtained from ACROS Organics. Dimethylurea was supplied by San-Apro Ltd. (Nishikyo-ku, Kyoto, Japan). Epoxy resin (Araldite^®^ GY 257) and phenyl glycidyl ether were provided by Huntsman Advanced Materials (The Woodlands, TX, USA). Araldite^®^ GY 257 is a low-viscosity bisphenol A-based epoxy resin modified with glycidyl ether, with a viscosity of 500–650 mPa·s at 25 °C and an epoxy value of 5.2–5.5 eq/kg. γ-glycidoxypropyltrimethoxysilane was purchased from Honeywell International Inc (Charlotte, NC, USA). A high temperature resistant thermoplastic polyurethane (TPU) substrate with a thickness of 100 μm was provided by San Fang Chemical Industry Co., Ltd. (Kaohsiung, Taiwan). All materials were used as received without further purification.

### 2.2. Preparation of Silver Conductive Adhesives

**Figure 1:**
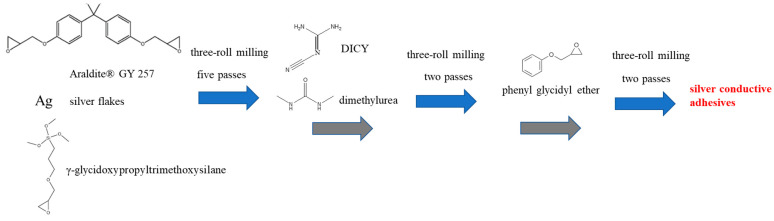
Schematic illustration of the preparation of silver conductive adhesives.

The preparation of the silver conductive adhesives is illustrated in Figure 1. In the preparation of the silver conductive adhesives, formulation components including epoxy (Araldite^®^ GY 257), silver flakes (SA-4391 or SA-4084), and a coupling agent (γ-glycidoxypropyltrimethoxysilane) were first mixed using a three-roll mill at a roller gap of 0.02 mm and a rotational speed of 50 rpm for five passes to achieve uniform dispersion. After the initial dispersion, the rotational speed was reduced to 25 rpm, and the curing agent DICY and accelerator (dimethylurea) were added, followed by two additional passes of three-roll milling. Finally, a diluent (phenyl glycidyl ether) was introduced and processed for another two passes to complete the compounding process. Phenyl glycidyl ether is a monofunctional reactive diluent. During curing, it participates in the epoxy ring-opening reaction and becomes incorporated into the crosslinked network rather than volatilizing. Because it contains only one epoxide group, its incorporation mainly reduces viscosity and may lower the effective crosslink density of the cured adhesive, while improving processability and conductivity. The organic carrier was composed of Araldite^®^ GY 257 (70.1 wt%), phenyl glycidyl ether (18.7 wt%), γ-glycidoxypropyltrimethoxysilane (4.7 wt%), DICY (3.7 wt%), and dimethylurea (2.8 wt%). The formulations of the conductive adhesive in this study were designed according to commonly reported silver-filled epoxy conductive adhesive systems [35,36,37].

### 2.3. Measurements

The thixotropic index (TI) was determined using a rotational viscometer (Brookfield Engineering Laboratories, Inc., Middleboro, MA, USA) at 25 °C. Viscosity was first measured at a low rotational speed of 0.5 rpm, followed by measurement at a high speed of 5 rpm; the ratio of these two values was defined as the TI.

Two parallel strips of 3M Miracle Scotch Tape were applied onto a standard 2.54 cm × 7.62 cm glass slide with a spacing of 0.254 cm. The test adhesive was then dispensed into the gap between the two tape strips. Using a single-edge razor blade held at a 45° angle, the excess adhesive was removed to form a uniform thin layer between the tapes; the blade edge was kept in contact with the tape to ensure a single smooth coating pass. The tape-adhesive-coated slide was then placed in a preheated oven and cured at 155 °C for 20 min. After curing, the tape was removed. The electrical resistance of the cured adhesive film was measured using a four-point probe system. It should be noted that the four-point probe measures surface resistance, which can be converted to volume resistivity based on the film thickness and electrode geometry.

Thermal conductivity was measured using a Hot Disk TPS 2500S instrument (Hot Disk AB, Göteborg, Sweden) based on the transient plane source (TPS) technique.

The optimal curing temperature was determined by differential scanning calorimetry (DSC) analysis. DSC analysis was conducted using a TA Instruments Q2000 calorimeter (TA Instruments, New Castle, DE, USA) under nitrogen flow. About 8–10 mg of the samples were heated from 40 °C to 240 °C at a heating rate of 10 °C/min and subsequently cooled to 40 °C at the same rate.

The morphologies of the silvers were examined using SEM (Hitachi-S4700, Hitachi, Tokyo, Japan).

The die shear strength of the silver conductive adhesive was evaluated using two chip sizes of 228.6 µm × 228.6 µm and 1143 µm × 1143 µm. Each die was bonded onto two types of substrates: (i) gold-plated aluminum nitride (AlN) substrates and (ii) TPU substrates featuring screen-printed silver electrodes. The silver conductive adhesive was dispensed onto the substrates, followed by die placement and curing under standard processing conditions. Die shear strength measurements were performed using a Nordson DAGE 4000 bond tester in accordance with the MIL-STD-883, Method 2019 [38] “Die Shear Strength” procedure.

### 2.4. GenAI

ChatGPT (version 5.0) was used to generate schematic illustrations to enhance clarity and improve reader understanding of the manuscript. The computer code used is not available.

## 3. Results and Discussion

Conventional silver epoxy adhesives typically require curing temperatures above 170–180 °C to achieve sufficient crosslinking, while low temperature curable systems often suffer from reduced storage stability and shortened pot life due to increased reactivity at ambient conditions [39,40,41,42]. In this context, the present study aims to address this trade-off by employing a DICY-based latent curing system, enabling both low temperature curing and extended pot life. Understanding formulation–property relationships was essential to simultaneously optimize conductivity, rheology, and application-specific requirements such as TI for flexible electronics, while enabling systematic studies on silver type and morphology. In practice, formulation must balance volume resistivity, thixotropy, and cost, as demonstrated by four experimental formulations (Table 1 and Table 2). Formulations 1 and 2 employ the same type of high conductivity silver flakes, and the total proportion of the organic carrier is kept constant across all formulations. Formulation 1, containing 75 wt% silver flakes (SA-4391) with a TI of 5.3, achieved a low volume resistivity of 9 × 10^−6^ Ω·cm and a thermal conductivity of 2.2 W/m·K, making it suitable for dispensing applications. In contrast, formulation 2 reduced the silver (SA-4391) content to 65 wt%, resulting in a higher volume resistivity of 1 × 10^−4^ Ω·cm and a lower thermal conductivity of 1.2 W/m·K, offering a cost-effective option for low power electronic applications. The high conductivity silver flakes used in these two formulations reached their maximum workable loading at approximately 75 wt%; further increasing the filler content leads to poor wetting and incomplete dispersion of the silver flakes within the organic carrier.

**Figure 2:**
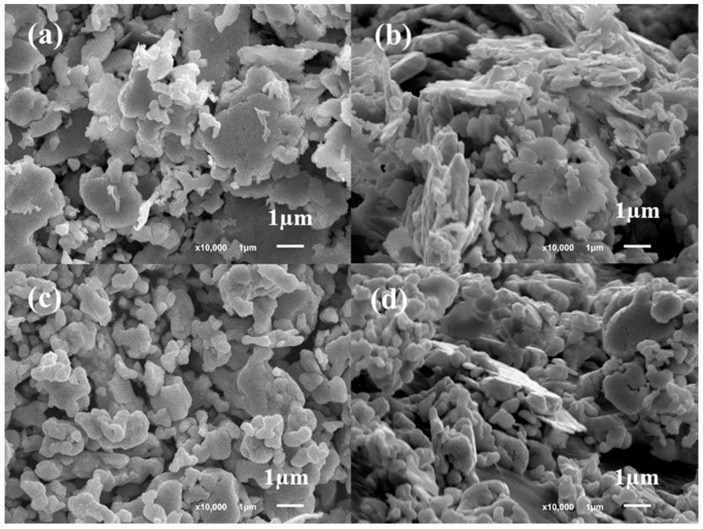
(**a**,**b**) SEM images of silver (SA-4391) used in formulation 1 and formulation 2 and (**c**,**d**) SEM images of silver (SA-4084) used in formulation 3 and formulation 4.

Formulation 3, incorporating high tap density silver flakes, was able to reach a filler loading of up to 85 wt% within the same organic carrier system. This is because the high tap density flakes possessed a smaller specific surface area than the high conductivity silver flakes (SA-4391) used in formulations 1 and 2 (Figure 2), allowing more efficient packing and requiring less organic vehicle for proper wetting. As a result, formulation 3 exhibited excellent electrical performance (1 × 10^−5^ Ω·cm) and a high thermal conductivity of 4.9 W/m·K, making it suitable for high power device applications, albeit at a higher material cost. Formulation 4 also utilizes the same high tap density silver flakes (SA-4084); however, its silver content was adjusted to match that of formulation 1. Despite having comparable thermal conductivity, formulation 4 showed a relatively higher volume resistivity of 8 × 10^−5^ Ω·cm, highlighting the critical role of silver morphology and filler loading in establishing an effective conductive network. Interestingly, despite its lower silver content, formulation 1 exhibited superior conductivity due to its large area, thin silver flake morphology facilitating efficient electron pathways (Figure 2). In contrast, the high tap density silver flakes used in formulation 3 and formulation 4 were relatively smaller in lateral size and thicker than the flakes in formulations 1 and 2 (Figure 2). In addition to overall filler loading, the electrical resistivity is strongly governed by particle–particle junction resistance, which typically dominates the conduction pathway in flake-based isotropic conductive adhesives. Large, thin flakes used in formulations 1 and 2 create broad interfacial contact areas, reducing constriction resistance and promoting efficient electron tunneling across flake interfaces. In contrast, the high tap density flakes in formulations 3 and 4, while offering superior packing density, exhibit smaller lateral dimensions and lower aspect ratios, resulting in reduced contact area per junction and therefore higher intrinsic interface resistance. These differences in junction resistance explain why formulation 1 achieves a lower resistivity (9 × 10^−6^ Ω·cm) than formulation 4 (8 × 10^−5^ Ω·cm), even at similar silver loadings. This highlights that conductivity is not solely determined by filler volume fraction, but also by the microscopic quality of interparticle junctions.

**Figure 3:**
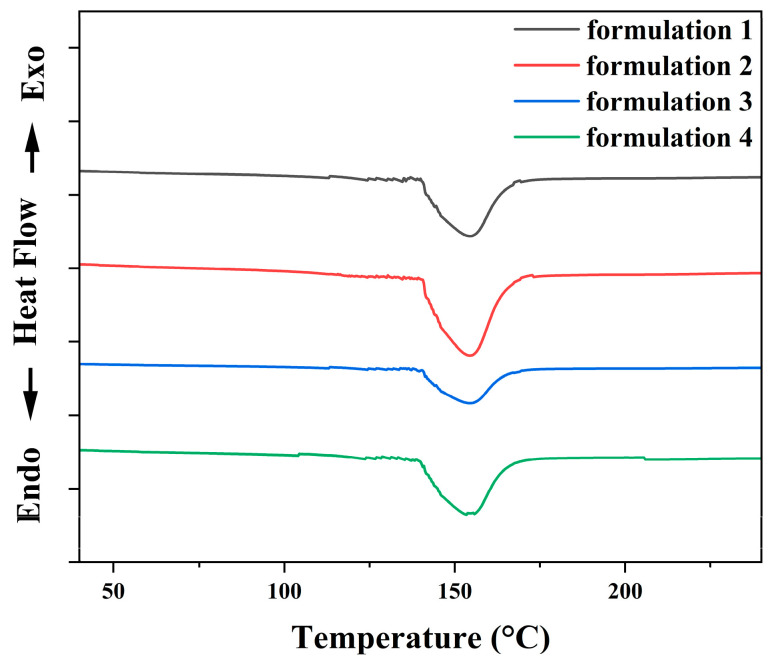
DSC curves of all formulations at the heating rate of 10 °C/min, under a nitrogen atmosphere. The *y*-axis scale corresponds to 10 mW per division.

**Figure 4:**
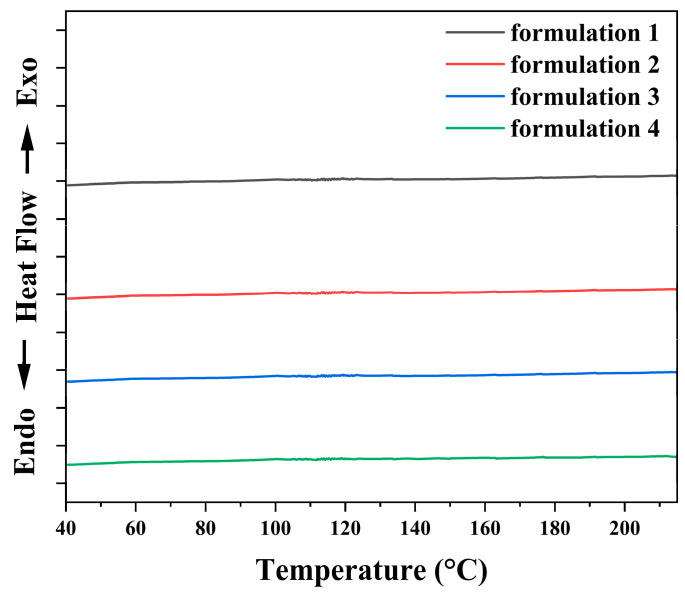
DSC curves of all formulations at the cooling rate of 10 °C/min, under a nitrogen atmosphere. The *y*-axis scale corresponds to 1 mW per division.

Figure 3 shows the DSC thermogram of all formulations at a heating rate of 10 °C/min. The curing reaction of the silver adhesive occurred between 145 °C and 160 °C, indicating that the optimal curing temperature was approximately 155 °C. The optimal curing temperature for formulations 1, 2, 3, and 4 was also around 155 °C because the relative proportions of epoxy resin, curing agent, accelerator and additives within the organic carrier were the same in each formulation. The low temperature curing behavior (~155 °C) originates from the use of a DICY latent curing system, which remains inactive at ambient conditions but becomes effectively activated upon heating. The effects of silane-mediated interfacial regulation of silver fillers suppressed premature reactions, enabling complete curing without the need for elevated temperatures or post-annealing processes. Figure 4 presents the DSC cooling curves of all formulations after the heating process, recorded at a cooling rate of 10 °C/min. No apparent exothermic or endothermic peaks were observed during cooling, indicating the absence of residual curing reactions or phase transitions. This result confirms that the curing process was essentially complete during the initial heating stage, demonstrating that full crosslinking of the adhesives was achieved under the selected curing conditions.

**Figure 5:**
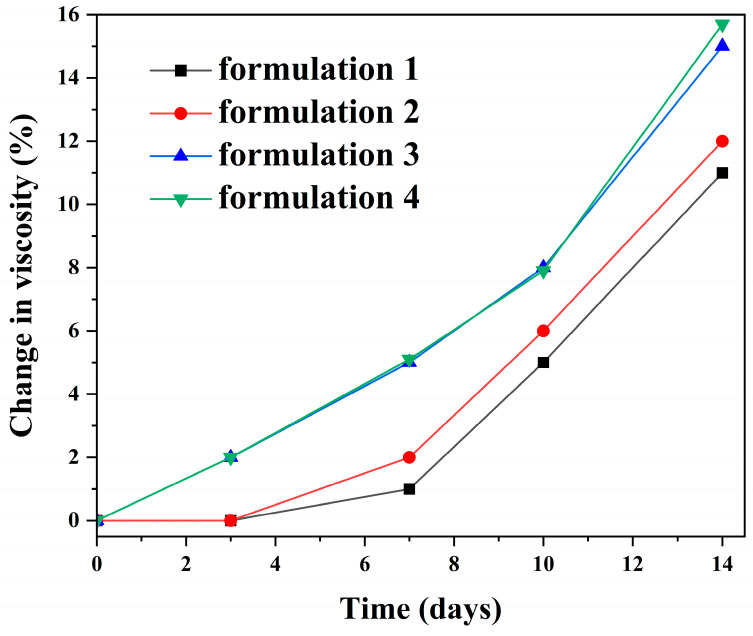
Change in viscosity (%) of the four silver adhesive formulations over time during storage at 25 °C and 60% relative humidity.

After being stored under an atmosphere with a relative humidity of 60% and the temperature of 25 °C for two weeks, the viscosity variation in formulations 1, 2, 3, and 4 remained within 16%, indicating that all silver adhesive formulations exhibited good pot life (Figure 5).

**Figure 6:**
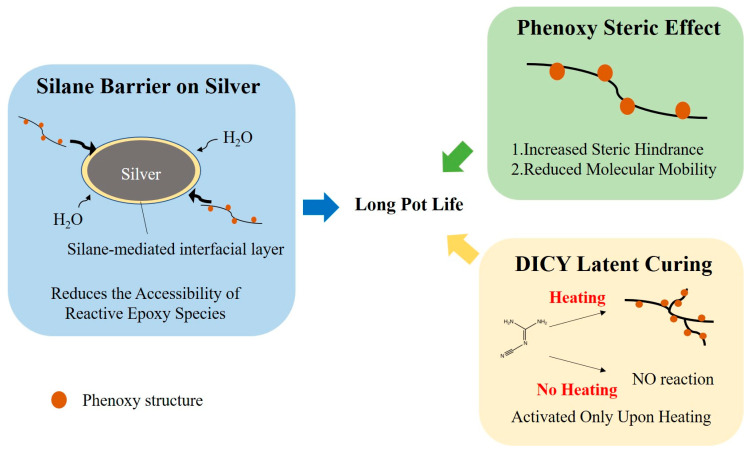
Schematic illustration of the long pot life mechanism.

A formulation was considered to retain good pot life when no abrupt viscosity increase, gelation, or loss of processability was observed within the evaluation period. In commercial conductive silver adhesives, datasheets commonly specify pot life/working life at room temperature ranging from minutes to days depending on whether the material is 2-part mixed or 1-part thawed (e.g., EPO-TEK^®^ H20E: 2.5 days; Henkel LOCTITE ABLESTIK C 805-1: 1–1.5 days; Henkel LOCTITE ABLESTIK 3888: 90 min; MG Chemicals 8331: 10 min working time) [39,40,41,42]. Although the definitions differ, our low viscosity drift over two weeks provides evidence of favorable storage robustness compared with typical room temperature usable windows specified for commercial formulations. The stability mainly came from using DICY as a latent curing agent, which has very low reactivity at room temperature and only cures upon heating, thus preserving viscosity [43,44,45]. In addition to DICY latent curing chemistry, the silane coupling agent further contributed to the suppression of pre-curing reactions. The phenoxy-containing structure (from Araldite^®^ GY 257) may introduce steric effects around epoxy groups and reduce molecular mobility, which could help limit unintended interactions between epoxy and accelerator species at room temperature [46,47,48]. The silane coupling agent grafted on silver surfaces forms a thin organosiloxane interphase that reduces the accessibility of reactive epoxy species and trace moisture to metal-associated interfacial sites, thereby delaying premature interfacial epoxy activation during storage. Together, these components provide physical and chemical stabilization of the epoxy–silver interface, preventing moisture-assisted reactions and significantly extending the shelf stability of the adhesive. Their synergistic effects complement the thermal latency of DICY, resulting in the excellent pot life performance observed for all formulations, as schematically illustrated in Figure 6.

**Figure 7:**
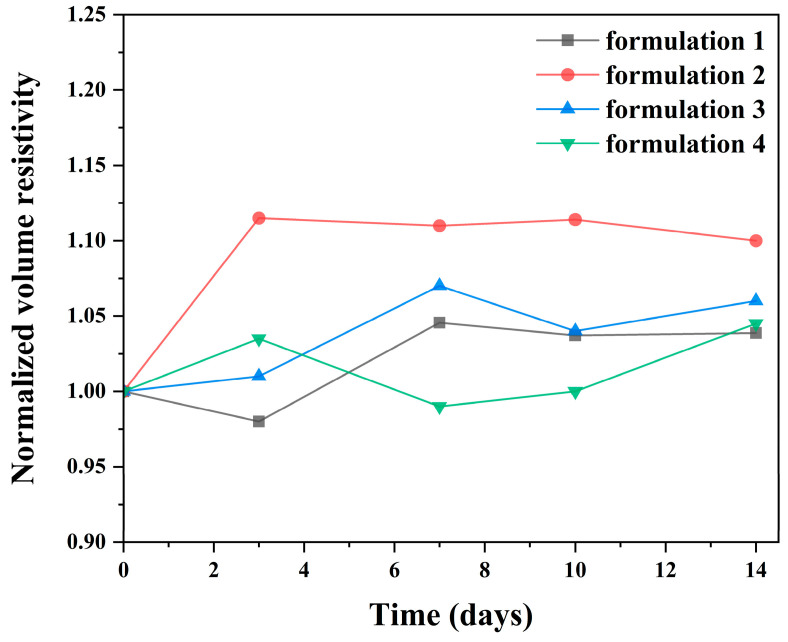
Normalized volume resistivity of the four silver adhesive formulations over time during storage at 25 °C and 60% relative humidity.

**Figure 8:**
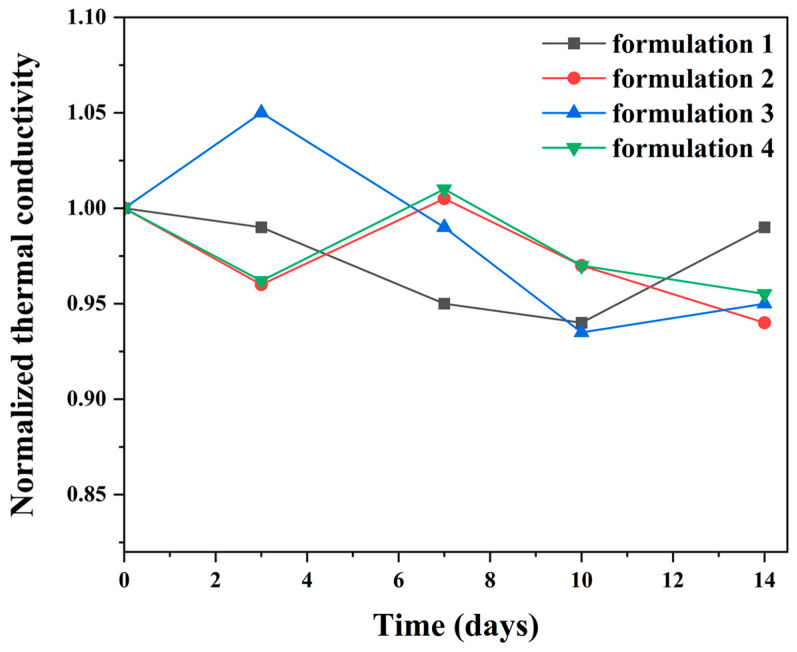
Normalized thermal conductivity of the four silver adhesive formulations over time during storage at 25 °C and 60% relative humidity.

To further elucidate the storage stability of the silver adhesive formulations beyond rheological behavior, the evolution of their electrical and thermal transport properties after long-term storage was systematically evaluated. After storage at 25 °C and 60% relative humidity for up to two weeks, the normalized volume resistivity of all formulations remained close to their initial values, with maximum relative deviations limited to approximately 12% compared to the Day 0 values (Figure 7). All formulations exhibited resistivity changes within the same order of magnitude, indicating a high level of electrical stability during storage. No monotonic increase or abrupt rise in resistivity was observed throughout the evaluation period, suggesting the absence of premature network formation, filler agglomeration, or moisture-induced degradation. These results demonstrated that the conductive pathways formed by the silver fillers remain well preserved under ambient storage conditions. Similarly, the normalized thermal conductivity of all formulations showed only modest changes over the two weeks storage period (Figure 8). The maximum relative deviation in thermal conductivity was limited to approximately 7% relative to the initial values, with all formulations remaining within a comparable and narrow variation range. Although minor fluctuations were observed at intermediate storage times, no systematic degradation trend was detected. This behavior indicated that interfacial thermal transport between the silver fillers and the epoxy matrix remained stable and was not significantly affected by moisture uptake or interfacial aging during storage. The simultaneous stability of viscosity, electrical resistivity, and thermal conductivity highlights the effectiveness of the formulation strategy.

**Figure 9:**
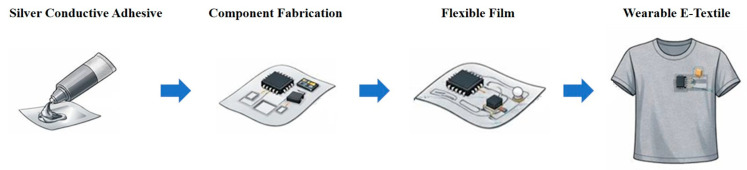
Schematic illustration of the application workflow of the silver conductive adhesive for E-textile integration, showing the fabrication of electronic components on a flexible film using the silver conductive adhesive and the subsequent transfer of the component-integrated film onto textile substrates for wearable applications.

Figure 9 shows the schematic illustration of the application workflow of the silver conductive adhesive for E-textile integration, showing the fabrication of electronic components on a flexible film using the silver conductive adhesive and the subsequent transfer of the component-integrated film onto textile substrates for wearable applications. The die shear force of the silver conductive adhesive was evaluated using 228.6 µm × 228.6 µm and 1143 µm × 1143 µm chips, which were bonded onto two types of substrates: gold-plated AlN substrates and TPU substrates featuring screen-printed silver electrodes. The silver adhesive was applied by dispensing, followed by curing under standard processing conditions. Die shear testing was conducted in accordance with MIL-STD-883, Method 2019. All silver adhesive samples successfully met the 2X level (highest-grade) requirement without die detachment on either rigid AlN or flexible TPU substrates, demonstrating robust interfacial adhesion and mechanical reliability. Detailed quantitative die shear force data for different formulations, die sizes, and substrates are summarized in Table 3. Adhesion strength is influenced by the organic phase fraction within the silver adhesive, with higher organic content favoring improved wetting and interfacial bonding. These results highlight the suitability of the developed silver adhesive for wearable electronics and flexible electronic devices. Overall, future development trends point toward the integration of multi-dimensional fillers to achieve high performance, low cost, and sustainability in conductive adhesives for next-generation wearable electronics and energy devices.

**Figure 10:**
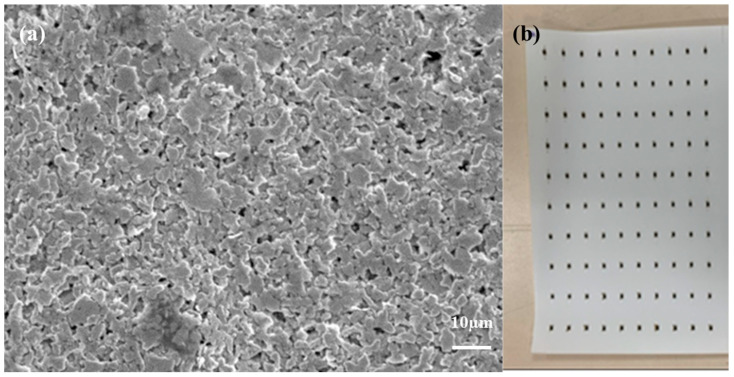
(**a**) SEM image of the fractured surface of the fully cured silver conductive adhesive prepared using formulation 1, showing a densely packed and well-dispersed silver filler network within the epoxy matrix. (**b**) Photograph showing the bonding of 1206-sized chip resistors onto a TPU substrate using the developed silver conductive adhesive based on formulation 1.

Figure 10 presents the microstructural characteristics and practical applicability of the developed silver conductive adhesive using formulation 1. As shown in Figure 10a, the SEM image of the fractured surface reveals a densely packed and well-connected silver filler network embedded within the epoxy matrix. The silver flakes are uniformly dispersed, forming continuous conductive pathways that are essential for achieving stable electrical conductivity. No significant agglomeration or large voids are observed, indicating effective filler–matrix interaction and good dispersion quality. In addition, Figure 10b demonstrates the practical bonding performance of the adhesive, where 1206-sized chip resistors are successfully attached onto a TPU substrate. The adhesive exhibits good wettability and adhesion, enabling reliable component fixation without noticeable misalignment or detachment. Furthermore, electrical resistance measurements revealed a high functional yield of 98%, indicating excellent electrical reliability and process stability.

## 4. Conclusions

This work establishes a formulation-guided design framework for epoxy-based silver conductive adhesives that integrates silver filler architecture with a DICY latent curing chemistry to achieve low temperature curability, long pot life, and balanced electrical–thermal performance. By systematically varying silver morphology and loading under a consistent organic carrier and curing framework, clear structure–property relationships were identified. Large area, thin silver flakes facilitated efficient electron transport by improving interflake contact quality, leading to low volume resistivity, whereas high tap density silver flakes enhanced thermal transport through improved packing efficiency. All formulations exhibited effective curing at a relatively low temperature (~155 °C), enabling a reduced thermal budget that is compatible with temperature-sensitive flexible polymer substrates. Importantly, the formulations demonstrated robust storage stability under ambient conditions. After storage at 25 °C and 60% relative humidity for two weeks, viscosity drift remained within ≤16%, and maximum relative deviations in normalized volume resistivity and thermal conductivity were limited to approximately 12% and 7%, respectively, indicating that rheological processability and functional transport pathways were well preserved during storage. Notably, these performance attributes were achieved without high-temperature sintering or post-annealing, highlighting the practical value of the latent-curing strategy and interfacial stabilization approach for manufacturable conductive adhesives. Overall, the results define a practical formulation design window to co-optimize low temperature curing capability, pot life, electrical conductivity, and thermal transport in epoxy–silver conductive adhesives, providing materials-level guidelines for conductive interconnects on flexible polymer substrates and related flexible electronic applications. Future work will focus on incorporating multidimensional hybrid fillers and conducting deformation- and environment-relevant reliability evaluations to further strengthen performance and application robustness.

## Figures and Tables

**Table 1:** Composition of the four silver adhesive formulations.

Materials	Formulation 1	Formulation 2	Formulation 3	Formulation 4
Silver content (%) ^a^	75	65	85	75
The total amount of organic carrier (%) ^b^	25	35	15	25

^a^ Formulations 1 and 2 use the same silver morphology (SA-4391), while formulations 3 and 4 use another identical type (SA-4084). ^b^ The relative proportions of epoxy resin, curing agent, accelerator and additives within the organic carrier are the same in each formulation.

**Table 2:** Properties of the four silver adhesive formulations.

Properties	Formulation 1	Formulation 2	Formulation 3	Formulation 4
TI	5.3	6.2	5.3	5.0
Volume resistivity (Ω·cm)	9 × 10^−6^	1 × 10^−4^	1 × 10^−5^	8 × 10^−5^
Thermal conductivity coefficient (W/m·K)	2.2	1.2	4.9	2.3
Optimal Curing Temperature (°C)	155			

**Table 3:** Shear force of different silver adhesive formulations for attaching chips of different sizes on various substrates.

Chips of Different Sizes/Substrates	Formulation 1	Formulation 2	Formulation 3	Formulation 4
228.6 µm × 228.6 µm chip on gold-plated AlN	1.82 kgf	3.42 kgf	0.98 kgf	1.78 kgf
1143 µm × 1143 µm chip on gold-plated AlN	over 5.00 kgf ^a^	over 5.00 kgf ^a^	4.02 kgf	over 5.00 kgf ^a^
228.6 µm × 228.6 µm chip on TPU	0.89 kgf	1.64 kgf	0.41 kgf	0.82 kgf
1143 µm × 1143 µm chip on TPU	3.71 kgf	over 5.00 kgf ^a^	1.98 kgf	3.70 kgf

^a^ Shear force was greater than 5.00 kgf.

## Data Availability

Data is contained within the article.
